# Velocity and Color Estimation Using Event-Based Clustering

**DOI:** 10.3390/s23249768

**Published:** 2023-12-11

**Authors:** Xavier Lesage, Rosalie Tran, Stéphane Mancini, Laurent Fesquet

**Affiliations:** 1 Univ. Grenoble Alpes, CNRS (National Centre for Scientific Research), Grenoble INP (Institute of Engineering), TIMA (Techniques of Informatics and Microelectronics for Integrated Systems Architecture), F-38000 Grenoble, France; xavier.lesage@univ-grenoble-alpes.fr (X.L.); rosalie.tran@univ-grenoble-alpes.fr (R.T.); stephane.mancini@univ-grenoble-alpes.fr (S.M.); 2 Orioma, F-38430 Moirans, France

**Keywords:** low-power tracking, event-based processing, embedded sensing, event polarity, event intensity, velocity estimation, color estimation

## Abstract

Event-based clustering provides a low-power embedded solution for low-level feature extraction in a scene. The algorithm utilizes the non-uniform sampling capability of event-based image sensors to measure local intensity variations within a scene. Consequently, the clustering algorithm forms similar event groups while simultaneously estimating their attributes. This work proposes taking advantage of additional event information in order to provide new attributes for further processing. We elaborate on the estimation of the object velocity using the mean motion of the cluster. Next, we are examining a novel form of events, which includes intensity measurement of the color at the concerned pixel. These events may be processed to estimate the rough color of a cluster, or the color distribution in a cluster. Lastly, this paper presents some applications that utilize these features. The resulting algorithms are applied and exercised thanks to a custom event-based simulator, which generates videos of outdoor scenes. The velocity estimation methods provide satisfactory results with a trade-off between accuracy and convergence speed. Regarding color estimation, the luminance estimation is challenging in the test cases, while the chrominance is precisely estimated. The estimated quantities are adequate for accurately classifying objects into predefined categories.

## 1. Introduction

Event-based image sensors (EBIS) are a new type of sensor that overcome some of the limitations of frame-based systems. Instead of capturing an entire image frame at a fixed rate, each pixel of event cameras responds to local changes in intensity, resulting in non-uniform sampling of the image and the suppression of temporal redundancies in the data. The resulting data stream is composed of events, each one encoding the information of the observed change. Such sensors require dedicated algorithms to exploit their properties, such as high temporal resolution and wide dynamic range, but also reduced data throughput, which is suitable for embedded systems.

Event-based algorithms have been developed for many vision tasks, from feature extraction to pattern recognition [1]. In a previous work [2], we proposed an event-by-event clustering algorithm that can retrieve the position and size of objects directly from the event stream of a Dynamic Vision Sensor (DVS) camera. With low-power sensing in mind, this work focuses on the low computational cost and small memory footprint of clustering.

In the literature on event-based algorithms, many algorithms process the classical DVS data stream by exploiting the position and the timestamp of the events [3,4]. This information is sufficient to perform many vision tasks [1,5], such as object tracking, optical flow estimation, and more. However, these methods often require the accumulation of many events to replace the missing intensity measurements of conventional sensors. An alternative approach to enhance either the algorithm’s performance or the understanding of the scene is to incorporate intensity measurements from Time to First Spike (TFS) pixels into the DVS data stream [6,7].

However, direct integration of intensity measurements into the event-based algorithm is not widely used. Numerous algorithms are established on frame-based equivalents, and they do not entirely leverage the asynchronous event information. Thus, we put forward a low-level event-by-event algorithm that does not necessitate a high-level representation of event information.

An example of such an event stream can be found in the Color Event Dataset of Scheerlinck et al. [8], which generates events by combining a conventional camera with a DVS. It is the first available dataset of its kind. It contains a wide variety of scenes that take advantage of the event cameras’ characteristics to cover a good range of vision tasks, but the nature of the acquisition limits the possible applications, as the main purpose of this dataset is image reconstruction. Similarly to our case study, the N-MuPeTS dataset by Bolten et al. [9] focuses on tracking individuals with varied color characteristics. Color segmentation is applied to the frames of a conventional camera and the resulting labels are assigned to the event stream of a DVS camera, with each event being given a corresponding color.

Nonetheless, few studies have suggested a processing approach for more intricate events that convey supplementary information, compared to the DVS event stream. State-of-the-art event-based object detection techniques, like the research carried out by Mondal et al. [10], or optical flow estimation [11,12] are still confined to the most fundamental type of events. To the best of our knowledge, only Marcireau et al. [13] have proposed a methodology to cluster and track uniformly colored objects with an event-by-event algorithm.

Following these observations, the goal of this paper is to propose simple algorithms which can make use of the enriched event stream in order to estimate the velocity and color of the clusters, as a continuation of the previous clustering work. To achieve this, a sensor is modelled in a custom event stream simulator.

Regarding velocity, we propose two simple and lightweight methods to estimate the speed and direction of clusters using optical flow assumptions. Both methods are compared and applied to a real video event stream. Then, we propose a method to estimate the color of an object from an enriched event stream under some assumptions. This rough estimate can then be used to classify the object color.

The paper is organized as follows: Section 2 presents the related works of event-based image sensors and introduces the case study as well as a model of the simulated sensor; Section 3 presents the state of the art of event simulators and the custom simulator along with the datasets; Section 4 provides the prior knowledge of spatio-temporal clustering required to fully understand the purpose of this work; Section 5 and Section 6 then detail the proposed methods for velocity and color estimation; and Section 7 discusses the results to conclude.

## 2. Event-Based Image Sensors

An event-based image sensor (EBIS) provides an alternative to conventional cameras that capture frames at regular time intervals. This type of sensor comprises self-triggering pixels that respond to local, relative changes in intensity. As a result, the throughput, and therefore the power consumption of the sensor, is significantly reduced. Since the scene is only partially sampled, the output depends both on the activity of the scene and the camera motion.

For further details on DVS pixels, we refer the interested reader to [3], and for TFS pixels, readers may refer to [7].

### 2.1. Operation Principle

The most common EBIS is the DVS [3], where each pixel is composed of two main components: a measuring element (such as a photodiode), which continuously captures the light intensity, and a change detector composed of a logarithmic differentiator circuit to measure the difference in luminance (logarithm of the intensity value) between two samples. Whenever this variation crosses a positive (resp. negative) threshold θ in the comparator circuit, an ON (resp. OFF) event is output with the corresponding polarity. After that, the pixel is reset and the process is repeated. Thus, a DVS event consists of time, position (address of the pixel), and polarity (sign of the change) information.

Additional luminance information can be generated by combining a DVS pixel with an element that measures intensity. One solution is to combine a conventional camera with Active-Pixel Sensor (APS) pixels with a DVS to form a Dynamic and Active Pixel Vision Sensor (DAVIS) [14], but the resulting sensor is not fully event-based. Another fully event-based solution is to combine a DVS with Time to First Spike (TFS) pixels, as proposed in the Hybrid DVS + TFS Pixel [7] and the Asynchronous Time-based Image Sensor (ATIS) [15]. These sensors work as follows: whenever the DVS circuit outputs an event (i.e., a significant change has been detected), the TFS circuit starts its integration to measure the current light intensity. It outputs a second event to indicate the end of the integration. Given these pairs of event spikes, a Time-to-Digital Converter (TDC) is used to measure the TFS time integration and to convert it to a digital value representing the intensity. Thus, the TDC step determines the resolution of the gray levels, and its depth determines the maximal integration time.

**Figure 1:**
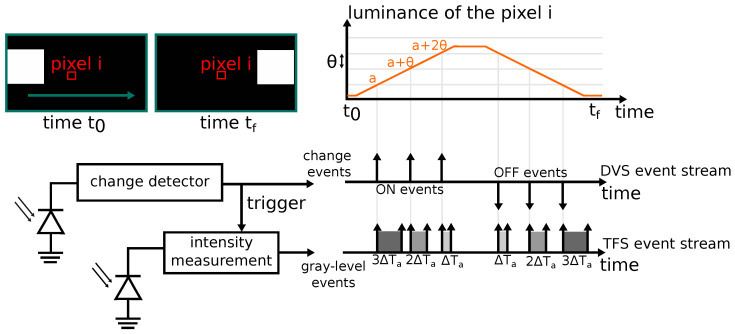
Operation of a hybrid (DVS + TFS) camera system: the change detector triggers the luminance intensity measurement; two types of event are then produced: change events indicating the increase/decrease in luminance, and gray-level events representing the intensity value.

The working principle of a hybrid DVS + TFS pixel is illustrated in Figure 1: Consider a white square moving towards the right side on a black background, passing in front of the pixel i. During this scene, the luminance profile of the pixel is similar to a step function, whenever it crosses the DVS threshold. Then, a DVS event is generated and triggers the TFS intensity measurement of the current luminance at the pixel i. As the square passes by, the generated events show the progressive integration of the intensity. The brighter the measured intensity, the shorter the integration time.

This concept of a change detector coupled to an intensity measurement can be extended to combinations of multiple change detectors and several intensity measurements, in particular an intensity measurement for each color channel. In this paper, a modelled sensor with three measuring elements, one for each of the red–green–blue (RGB) components and one sole change detector, is simulated. For the sake of simplicity, the three elements are assumed to be stacked on top of each other at each pixel, so that the color information retrieved at that location is grabbed from the same pixel at the same time, which is not the case for sensors using a Bayer filter array (see Section 7.1). The RGB values are then linearly combined to obtain the luminance component, which is input to the change detector. The color representation in the algorithm is the YCbCr format, which separately represents luminance and chrominance. The Y channel represents a corrected luminance that mimics the sensitivity of the human eye, with its specific sensitivity for each primary color. The Cb and Cr channels represent color values in a dichromatic plane, based on the blue difference and red difference. The RGB components are converted to the YCbCr format.

The change and intensity events from the DVS and TFS streams are combined into events of the form: $ev=\{e_{x},e_{y},e_{t},e_{p},e_{lum},e_{cb},e_{cr}\}$, where $e_{x},e_{y}$ corresponds to the position, $e_{t}$ the timestamp, $e_{p}$ the polarity, and $e_{lum},e_{cb},e_{cr}$ the components in the YCbCr format.

### 2.2. Case Study

To leverage the modelled sensor’s capabilities, we make certain assumptions to ensure accurate estimation of the cluster velocity direction and color, as detailed later. This paper aims to present the primary estimation approaches in a clear and concise manner. We limit our use case scope to enhance comprehension, but the proposed methodology can be modified to alleviate these constraints.

- The camera should be stationary, so that events only depend on the motion in the scene, not on the camera motion. Otherwise, events can be generated by the background due to the camera displacement. A pre-processing step can help to distinguish the events involved in the movement of objects [16].
- The camera point of view should be a top-down view, looking down at the ground, so that the objects are supposed to be not or slightly deformable. To account for deformation, further processing of the clusters are required such as cluster fusion [17,18] or probability distributions that handle occlusions [19].
- The white light source should also be invariant in time and at the top, facing towards the ground, to avoid shadows outside the objects.
- The color of the objects and the background should be locally uniform (untextured) and monochromatic. Since events are produced on edges, a textured surface would produce events across its whole surface, resulting in an inaccurate estimation or requiring further processing.

The operation principle of the presented sensor is simulated by a custom event simulator, which is introduced in the next section.

## 3. Event-Based Simulators

In this section, state-of-the-art simulators are presented, as well as our custom event-based simulator.

### 3.1. Related Works

The DAVIS simulator [20] is based on an ideal DAVIS camera which, given a virtual 3D scene and the trajectory of a DAVIS camera, generates the corresponding event streams, intensity, and depth maps. It relies on a rendering software to render images that are used by the simulator to generate event streams with event interpolation.

The open event camera simulator (ESIM) [21] is an improved version of the previous simulator, as it is more complete and provides ground truth for camera pose, motion, depth, and optical flow. In addition, the camera parameters and noise generation can be simulated.

The v2e simulator [22] proposes to generate realistic event streams from conventional video frames by simulating the behavior of DVS pixels. It integrates a linear-to-logarithm conversion for intensity frames, as well as temporal noise event addition. An optional frame interpolation using the Super-SloMo network [23] is included to improve the accuracy, but the processing time can be very long.

Although both the DAVIS and ESIM simulators are powerful tools as they provide multiple ground truth information about the observed scene, they mostly attract interest for moving camera scenarios since they require camera trajectory and prior scene modelization. However, our case application (stationary camera) cannot be fully exploited with these two simulators, as they do not provide ground truth for object tracking or object velocity estimation. The v2e tool could be used, especially to improve the accuracy of the model for DVS events. However, these three simulators work with non-colored events, meaning that the color information needed in our algorithm is discarded and cannot be retrieved. This is a hindrance, as our algorithm works with both spatio-temporal and color information at the same time. The Color Event Dataset [8] presents the same problem as the simulators, as it does not fit the case of the stationary sensor study.

### 3.2. Custom Event-Based Simulator

**Figure 2:**
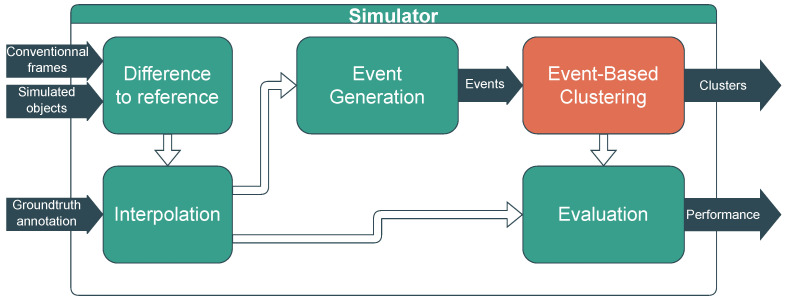
Overview of the simulator used: an event stream generator (green blocks) followed by the event-based clustering algorithm to test (orange block).

As a continuation of the previous work [2], the simulator was extended to include the TFS pixels. It consists of two parts (see Figure 2): an event stream generator, described below, and the design under test, in our case an event-by-event clustering algorithm (the orange block in Figure 2).

The event stream results from the translation of a conventional video, i.e., a sequence of frames. Events are generated by measuring the luminance difference between the current frame and a reference frame; if this difference exceeds a defined threshold, an event is generated. The reference frame is then locally updated with the crossed value. This is necessary to detect slowly changing pixels that require several frames to cross the amount of the threshold.

Unlike real EBIS, the sampling of the input video is uniform in time (fixed by the video frame rate), and it is required to apply an interpolation step to approximate the continuous quantity between frames. As a result, multiple events can be generated from the same pixel between two video frames if multiple thresholds are crossed meanwhile. The generated events are composed of the interpolated timestamp of the threshold crossing and the interpolated YCbCr values of the pixel.

## 4. Experimental Datasets

Several videos were recorded in order to test the algorithms with more realistic data. Two of them are presented below and summarized in the Table 1. The recording conditions are the same as those described in Section 2.2 to ensure proper functioning of the algorithms.

**Figure 3:**
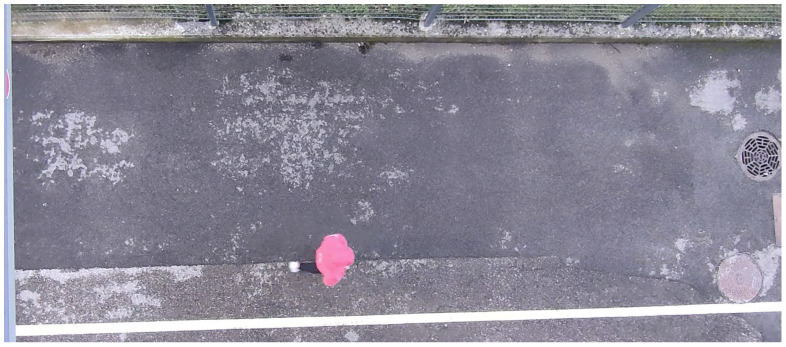
Example of a frame from the *Pedestrian* case (rotated sideways).

In the first video, a person with a pink sweatshirt can be seen walking at various speeds, along the vertical and the horizontal axes, as displayed in Figure 3. Note that the background has a texture and is not uniform, the person’s direction changes frequently, and the person is multicolored (as discussed later in the text). This dataset aims to assess velocity estimation across different trajectories and color estimation against textured backgrounds and non-uniform objects.

**Figure 4:**
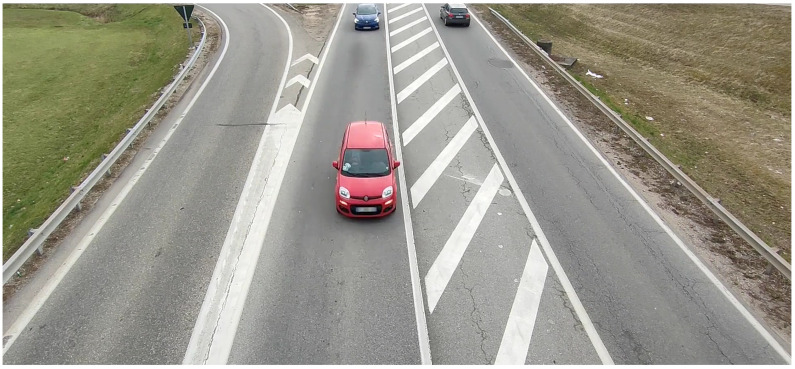
Example of a frame from the *Traffic* case.

The second video shows a sequence of 12 colored cars moving in two opposite directions along the vertical axis. A frame example can be seen in Figure 4. The speed and direction of each car are similar (in absolute value). A color class is assigned to each car from a predefined palette based on the car’s average color (including the colored parts of the image not belonging to the cars). Table 2 presents the distribution of car classes and their corresponding color. This dataset is primarily intended for evaluating the estimation of car velocities within a certain range, and for assessing color classification, particularly for color shades.

The videos are processed and manually annotated. Each object is defined by a rectangular bounding box, marked out as a pedestrian or a car, and by its color class. The annotated information (considered the ground truth) consists of the center of the box (position), the size, the color (average of the pixels considered to be part of the object), and the color label of each object in each frame. The ground truth velocity can be retrieved as a vector between two consecutive positions (centers) of an object.

The video frame rate is adjusted based on the speed of the objects. Interpolation helps in the modelling of an EBIS for slow-moving objects. However, the frame rate may need to be adjusted if there are fast-moving objects that travel a significant distance between successive frames. The ground truth color of each object is obtained through a basic color segmentation within its respective bounding box. The main colors, along with the background color, are provided to test the algorithm.

Since there is no simulator or dataset that provides similar events with ground truth information on velocity and color, the custom event simulator is used to generate color events for the clustering algorithm.

## 5. Spatio-Temporal Clustering

Section 3 offers an overview of event-based simulators, along with a model of the sensor proposed in this work. This section presents the event-by-event clustering algorithm, which forms the basis of the contributions made in this work.

Clustering consists of grouping data that are similar under one entity, so that data within the same cluster are more similar to each other than to any other data. In our case, the purpose is to identify and track objects of interest in the scene. This involves updating the properties of these objects while filtering out irrelevant clusters that do not correspond to any object in the scene.

The first step of clustering is to collect similar events under a group called a cluster. Typically, a cluster is defined by a set of attributes including its size, center, and activity (which measures the rate of events). Ideally, each cluster corresponds to an object to be tracked in the scene. However, noise can cause formation of unwanted clusters that need to be removed.

The second step involves updating the clusters by incorporating the latest scene information and computing new values for each attribute. Therefore, the tracking data solely comprise the attributes of each cluster and the information of the last event that took place.

### 5.1. Related Works

The event-based clustering technique began with Litzenberger et al.’s [24] event-by-event clustering algorithm. This technique relies on the mean-shift method, which iteratively shifts the center towards the maximal density increase to locate the maxima of a highly dense region. In other words, the mean information of events is extracted. In this algorithm, individual events, which are points in the spatio-temporal space, are processed before being discarded due to the iterative nature of the process, leading to the event-by-event property.

The work of Lagorce et al. [25] and the improvement by Aladem et al. [18] use bivariate Gaussian distributions to describe the event clusters, and the events are distributed in the cluster based on the maximum likelihood. Another example of such a clustering algorithm is the work of Barranco et al. [26] that uses Kalman filters to smoothen trajectories. These methods require expensive computations for cluster updates; thus, the simpler approach of Litzenberger et al. [24] is preferred, as well as the modifications proposed by Zhao et al. [17]. However, the primary distinction from previous studies [17,24] is the implementation of decaying activity that is essential to precisely estimate event rates. Further adjustments have been made, but they are beyond the scope of this paper.

### 5.2. Mean-Shift Principle

For each incoming event, the algorithm searches for the nearest cluster within its seeking range and then updates it. If the algorithm does not locate any cluster, it initializes a new one with the information of the event. Consequently, the only data preserved in memory are a list of all clusters, ordered according to their creation timestamp.

The cluster update is handled with the mean-shift approach. That is, every attribute *X* of the cluster is revised using the following formula:

(1) $$\begin{array}{c} X_{n}=\alpha_{X}X_{n-1}+\left(1-\alpha_{X}\right)f_{X}\left(ev\right) \end{array}$$

where *n* is the timestamp of the current event and n−1 is the timestamp of the latest event of the cluster; $\alpha_{X}\in \left[0,1\right]$ is the mean-shift parameter for the attribute *X*; and $f_{X}\left(ev\right)$ is the change brought by the event.

This is a recursive low-pass filter that is non-periodic, since the time difference between *n* and n−1 is not constant.

A single event does not provide enough information to be meaningful, but the accumulation of many events allows this method to efficiently track objects as clusters, or extract regions of interest. Periodic filtering of the list of clusters is performed based on the cluster activity to remove noise and inactive clusters.

**Figure 5:**
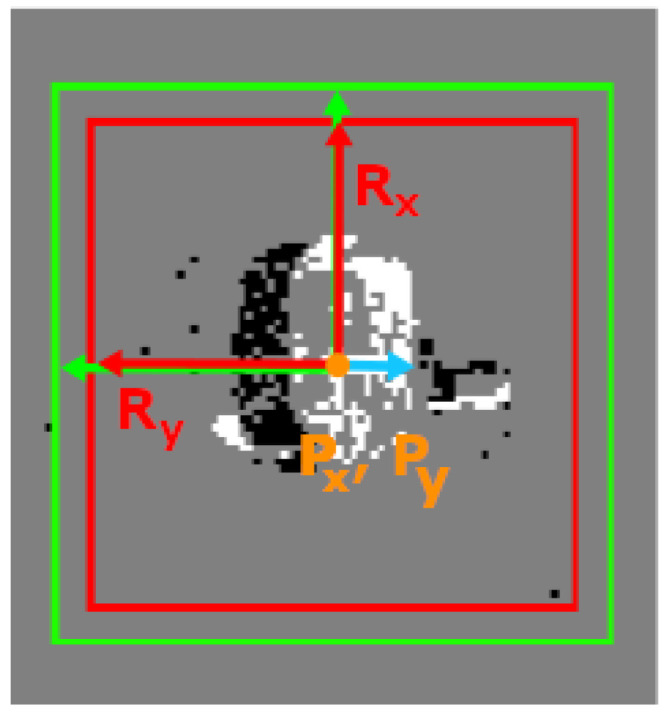
Example of a cluster with its attributes.

Table 3 presents the attributes of a cluster. The center position (i.e., the center of mass) and the size of the cluster are updated based on the incoming event’s position. The activity represents the rate of events assigned to the cluster. See also Figure 5 for a visual representation where the red box represents the cluster, the green box the seek region, the orange dot the center, and the blue arrow the velocity vector. This summarizes the implemented algorithm in the details required to understand the contribution; however, interested readers may refer to [2].

The basic clustering algorithm is sufficient for tracking objects and outputting regions of interest for the purpose of more complex processing. This work proposes the addition of a velocity attribute and a color attribute, which are estimated using similar methods.

## 6. Velocity Estimation

The velocity of the objects can be a helpful attribute to further improve object tracking. It allows trajectory estimation and motion segmentation. By knowing the velocity magnitude and direction of the objects, the tracking performance can be improved by filtering noise, detecting objects leaving the scene, and tracking motionless objects.

### 6.1. Related Works and Assumptions

The proposed methods are based on the assumptions made for estimating optical flow. Since only one motion vector is necessary for tracking an object, the velocity estimation method described in this section calculates the motion vector of the object’s center.

Classical optical flow methods aim to calculate the motion vectors for many points in the scene between two consecutive image frames. Specifically, two studies offer distinct constraints on the optical flow problem and establish the fundamental assumptions for various methods. The first study is the method of Lucas and Kanade [27], which identifies the best match between two images by minimizing the matching error for a limited number of points within a specified area of interest. It operates under the premise that local flow remains constant in a small neighborhood. The second study is the Horn and Schunck method [28], which assumes that the global flow is constant and smooth.

These assumptions can be modified to fit event-based systems. Therefore, algorithms designed for the classical framework can also be adjusted to process event data. In particular, Benosman et al. [29] assume the local optical flow constancy described by Lucas and Kanade [27] and apply it to a small spatio-temporal neighborhood around the examined pixel. The missing luminance function is replaced by accumulating events over time. Brosch et al. [11] noted that this approach may produce inconsistent outcomes due to approximations in the computations of the gradient, and the potentially low number of events. Using a consistent second derivative equation, Brosch et al. [11] proposed a further refinement of the gradient-based method. However, for more accurate flow estimation at the expense of higher computational costs, Brosch et al. suggest using an event plane in the spatio-temporal space or filter-based approaches. These methods are closer to biologically plausible systems.

However, the methods mentioned above rely on event buffering, which represents events integrated at a high level over a time duration. In Mueggler et al.’s research [30], the authors proposed a trajectory that updates continuously over time using information from each event. Similarly, Stoffregen et al. [12] presented a method for optical flow and segmentation, which uses the projection of events along the trajectory. Both of these methods are more general than the one presented here, because they also consider ego-motion.

The aforementioned clustering algorithm can incorporate a velocity estimation using the cluster property. The work of Aladem et al. [18] refers to utilize velocity, with a focus on angular estimation, when merging clusters that move in the same direction to create a larger, singular cluster. In this case, the velocity is estimated using the covariance matrix of the cluster representation. However, this paper does not present a quantitative result for the estimation error, and only shows the result in simple scenarios. The case study of Litzenberger et al. [31] is similar to ours and uses a level crossing scheme based on the distance crossed by the cars. This method relies on the constraints of the application, since the speed is directly estimated as the time to travel a given 1D distance.

This paper estimates velocity by calculating the motion of the center of mass of the object during the clustering phase. This estimation is an approximation of optical flow, since it is only computed for the cluster center. However, this implies that no rotation or deformation of the object, affecting the position of the center of mass, degrades the estimation. For this reason, it is assumed that the objects are rigid, even though their shape may change slightly due to perspective. The motion vector of the center is approximated as a translation, since the local optical flow is consistent [27]. In other words, it is postulated that events in the same spatio-temporal neighborhood contribute to the same translational motion (see [12,29]). Finally, the estimated velocity is the apparent velocity of the center. It does not correspond to the real motion of the object. Another function is required to translate this velocity, depending on the scene and camera setting.

### 6.2. Algorithms

This paper presents two methods for estimating velocity: the first approach calculates the change in cluster position at regular time intervals, while the second utilizes the mean-shift approach to estimate the average motion of the cluster center.

#### 6.2.1. Regular Time Interval Estimation

One of the most intuitive methods is to periodically compute the change in position. The time period between two estimations is used to assess the velocity using a mean-shift approach, which also helps to smooth the estimations.

The estimation is defined by the following equation:

(2) $${\hat{V}}_{RI}\left(t\right)=\alpha_{vel,RI}{\hat{V}}_{RI}\left(t-\tau \right)+\left(1-\alpha_{vel,RI}\right)\frac{P(t)-P(t-\tau )}{\tau }$$

where ${\hat{V}}_{RI}$ is the estimated velocity at regular estimation instants, $\alpha_{vel,RI}$ is the mean-shift parameter used to smooth the estimation, *P* is the position of the center of the cluster, and τ is the time period between two successive estimation instants.

To ensure proper operation of this method, the time period τ must be sufficiently long so that enough events occur during each time interval. It should be noted that macroscopic-level changes are only represented by the accumulation of events. Moreover, the parameter $\alpha_{vel,RI}$ is required due to the estimation’s high sensitivity to noise. Since these two parameters together act as a low-pass filter that eliminates noise, the same equations can be used to determine $\alpha_{vel,RI}$ from τ with appropriate noise characterization.

The velocity estimation can be done in parallel with clustering, analogous to the filtering. Therefore, ${\hat{V}}_{RI}\left(t\right)$ is a continuous function that can be sampled at any time (for any τ). Furthermore, just as with the filtering, it is possible to define other types of intervals. The proposed version employs a time interval, but spatial intervals [24,32] or a fixed number of events could also be utilized. However, no definitive results are presented in this version.

This approach provides an accurate and straightforward way to estimate velocity, although convergence can be slow due to the periodic estimation. Nevertheless, when objects produce sufficient events, the time period can be reduced, accelerating the convergence.

#### 6.2.2. Mean Center Motion (MCM)

A second method is proposed to enhance responsiveness, particularly for high-speed object tracking. This method relies solely on the position shift generated by each event within the mean-shift-based clustering.

From (1) we obtain the following:

(3) $$\begin{array}{cc} {\hat{P}}_{n} & =\alpha_{c}{\hat{P}}_{n-1}+\left(1-\alpha_{c}\right)\left(e_{x},e_{y}\right) \end{array}$$

(4) $$\begin{array}{cc} {\hat{P}}_{n}-{\hat{P}}_{n-1} & =\left(\alpha_{c}-1\right){\hat{P}}_{n-1}+\left(1-\alpha_{c}\right)\left(e_{x},e_{y}\right) \end{array}$$

(5) $$\begin{array}{cc} {\hat{V}}_{MCM,n} & =\alpha_{vel,MCM}{\hat{V}}_{MCM,n-1}+\left(1-\alpha_{vel,MCM}\right)\left({\hat{P}}_{n}-{\hat{P}}_{n-1}\right)\gamma \end{array}$$

(6) $$\begin{array}{cc} {\hat{V}}_{MCM,n} & =\alpha_{vel,MCM}{\hat{V}}_{MCM,n-1}+\left(1-\alpha_{vel,MCM}\right)\left[\left(\alpha_{c}-1\right){\hat{P}}_{n-1}+\left(1-\alpha_{c}\right)\left(e_{x},e_{y}\right)\right]\gamma \end{array}$$

where ${\hat{V}}_{MCM,n}$ is the estimated velocity at the timestamp of event *n*, $\alpha_{vel,MCM}$ is the velocity mean-shift parameter, $P_{n}$ is the center of the cluster at the timestamp of event *n*, and γ is a proportionality factor for the magnitude of the estimation.

In contrast to the previous method, this estimation is processed for each event, resulting in a more responsive estimation in time, but at the cost of more frequent computations. The term $\left(P_{n}-P_{n-1}\right)$ denotes the shift in the position of a single cluster caused by an event. Accordingly, the previous position is also stored in memory.

The parameter γ is a proportionality factor that scales the estimated velocity to the apparent object velocity (see (5)). It depends on the amount of events generated; therefore, the speed, the contour, and the size of the objects are the main factors influencing the value of γ. Furthermore, since the estimation is not calculated at regular time intervals, γ replaces the time interval. The parameter γ serves as an operating point for a given scene. In our applications, it is set such that the estimated velocities are comparable to the ground truth velocities. In the cases of multiple objects or missing ground truth, the operating point is set according to the mean velocity or expected velocity in the scene. If the speed of objects has a great variance, multiple operating points can be defined.

To further improve the results of this method, it is recommended to replace the parameter γ with a function that depends on the attributes of each cluster, which would improve the model’s ability to account for the amount of generated events. However, this requires more processing as it may require an estimation of the contour, similar to [12]. This approach provides better responsiveness in estimates at the cost of increased sensitivity to noise and parameter tuning.

### 6.3. Experiments and Results

The results of velocity estimation for the various experiments and other applications of velocity estimation are presented and analyzed in this section.

**Figure 6:**
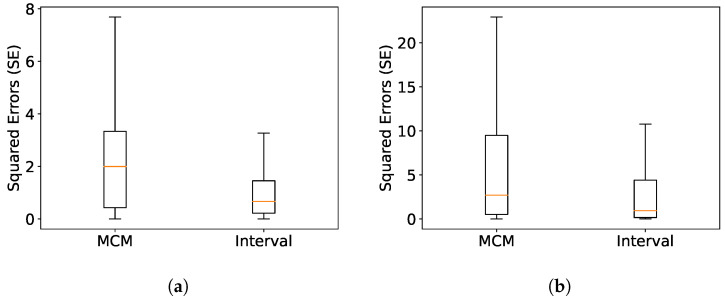
Box plot for velocity error. (**a**) Pedestrian; (**b**) Traffic.

The velocity estimation is evaluated using the root mean squared error (RMSE), which compares the estimated and true velocity vectors (see Section 4). Furthermore, a normalized value is given alongside using the mean ground truth value (normalized RMSE). Additionally, the mean angular error is determined using cosine similarity between the estimation and the corresponding ground truth. Both evaluations are performed at every filtering step (50–75 events), resulting in more evaluation points than frames. All velocity magnitudes are expressed in pixel per frame time, while angular errors are expressed in degrees. To optimize the parameters, a brief video segment was utilized and the hyperopt framework [33] was employed on this segment to optimize the parameters. Table 4 presents the optimized parameters for each scenario from 100 parameter sets, with the evaluation results provided in Table 5. Figure 6 shows box plots of the squared error in estimation. The orange line represents the median, and the whiskers encompass 3 quartiles of the error, without displaying outliers.

**Figure 7:**
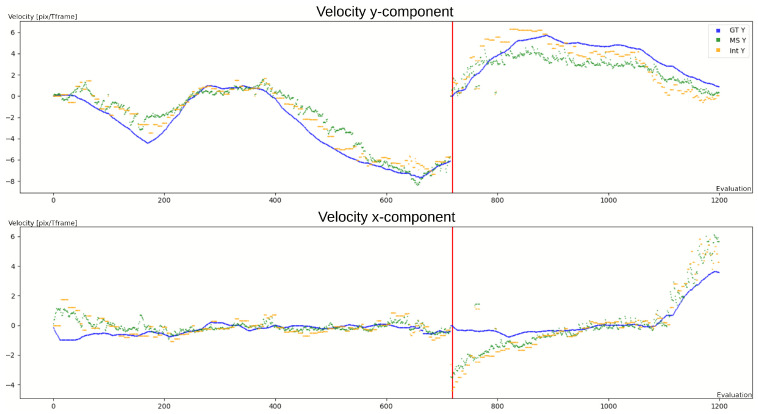
Tracking of one person in the *Pedestrian* case: (**top**) velocity y-component; (**bottom**) velocity x-component; ground truth is shown in blue; MCM estimation is shown in green; and regular interval estimation is shown in yellow. The red line indicates the object’s exit and re-entrance.

Figure 7 displays the estimated velocity in pixel/$T_{frame}$ for *Pedestrian*, using green for the mean center motion method and yellow for the regular interval method. The y-axis indicates the estimated velocity in pixel/$T_{frame}$, where $T_{frame}$ denotes the period between two frames. The x-axis represents the number of evaluations conducted. The object leaving and re-entering the scene later on is indicated by the red vertical line.

The optimization procedure results in $\alpha_{vel}$ parameters that are near 1, since many events are required to obtain accurate estimation of the motion. The choice of these parameters presents a trade-off between accuracy and responsiveness, depending on the estimation frequency. Figure 7 illustrates this point, as both estimations have a delay compared to the ground truth. The regular estimation time interval τ falls within the range of 1 $T_{frame}$ to 3 $T_{frame}$. This means that at maximum, the equivalent of three frames are required to accurately estimate the velocity from the events.

In general, the MCM approach presents a better responsiveness than the regular interval method, since it assesses the velocity more frequently than the latter; however, it is more sensitive to noise. The MCM method may have a higher error, but it depends mainly on the velocity scaling. On the other hand, the MCM method shows a slightly smaller angular error. This is reflected in the box plots, since the variance in error is lower for the interval method.

For both *Pedestrian* and *Traffic*, we conclude that the primary source of error is the object’s varying shape, especially upon scene entrance and exit. As a result, the methods converge only when objects are entirely within the scene (see steps 740–820 for the x component in Figure 7). This results in the upper whisker being far from the median in Figure 6.

Furthermore, the chosen error metric is sensitive to outliers that occur at the entrance before convergence.

In *Traffic*, the wider range of velocities leads to increased errors. However, straight trajectories result in fewer angular errors. In certain instances, cars move so swiftly that they only appear briefly in a few frames. In these scenarios, the simulator’s event generation is less precise compared to a real EBIS, leading to decreased accuracy in the methods.

In the experiments, the proportionality factor γ is constant and global. However, a better outcome can be obtained by replacing this constant gamma with a function that considers the scene and object characteristics to reconstruct the objects’ actual velocity in the scene. Fulfilling this goal necessitates exact estimation of each object’s properties alongside the camera position and angle. Moreover, each object’s characteristics are required to carry out precise estimation of the object velocity. One potential cause for inaccurate estimation is the conversion from videos (frame-based) that do not capture precise temporal information.

#### Applications with Velocity

In the following section, a few applications of the estimated velocity are presented.

The first application involves filtering noise clusters based on their velocity. Since the estimated velocity of noise is approximately zero, the noise pattern can be easily filtered out using one of the previously introduced methods. To achieve this, a velocity threshold is set below the expected minimum velocity value. This threshold is then compared to the estimated velocity of each cluster to determine whether the cluster is considered noise or not. This approach improves tracking performance by allowing the activity threshold to be lowered, resulting in more consistent tracking without introducing additional noise. In other words, reducing the recall–precision tradeoff by lowering the activity threshold can be achieved by using velocity filtering.

**Figure 8:**
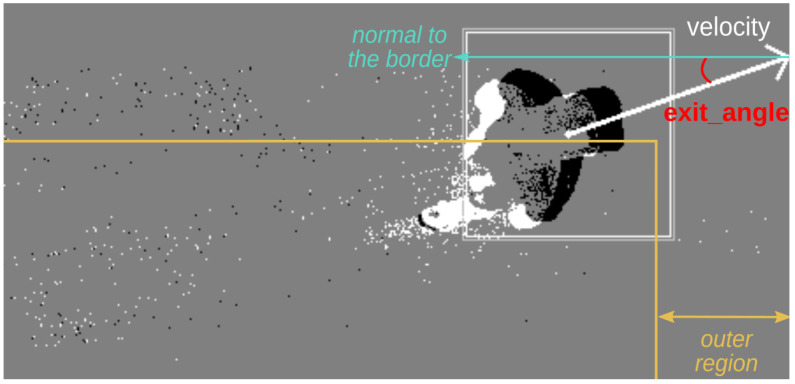
Example of a cluster about to exit the field of view of the sensor.

Moreover, the velocity direction can be used to detect when objects are about to leave the scene, allowing the removal of the corresponding clusters (see Figure 8). To begin with, the algorithm checks if the cluster is in the outer region of the scene, in other words, if it is close to one of the four borders. If the velocity direction points towards the borders in such a scenario, it is possible that the cluster is about to exit the scene. The final verification ensures that the velocity magnitude is sufficient and that the angle at which it exits is small enough (close to being perpendicular to the border).

To reduce the number of clusters that need to be evaluated, it is suggested to launch a filtering step before predicting the object exit, as this prediction adds a few more steps with conditional verifications to the clustering algorithm. As previously discussed, object entrance and exit cause discontinuities that generate non-negligible errors in velocity estimation. Therefore, detecting object exit based on the velocity direction of clusters increases the performance of the clustering algorithm. Similarly, an object’s entrance could be detected with appropriate modifications of the algorithm.

Both methods offer solutions to estimate cluster velocity with minimal extra computations. As we constrain ourselves to low-power, low-calculation applications, the outcomes are less accurate compared to the values cited in [12,30]. Neither [18] nor [24] report the angular error, but we consider an angular error of less than 20 sufficient for exit detection and the estimation of rough trajectory. Moreover, errors are primarily attributed to outliers in the error distribution. Despite the trade-off between accuracy and responsiveness, the methods perform well in multiple scenarios. Furthermore, velocity can improve the clustering process by filtering noise and detecting objects that exit the scene.

## 7. Color Estimation

A significant difference between EBIS and conventional image sensors is the difficulty in extracting color information from the event stream. Color and texture are inherent traits of mammalian vision, and are useful for complex tasks such as object recognition, where color is essential for distinguishing objects. Thus, retrieving color from the event stream enables color segmentation of the scene and object classification based on the color.

### 7.1. Related Works and Assumptions

Dedicated sensors are required to extract color from EBIS. Thus, many works have aimed to enrich the classical DVS [3] event stream, which only quantifies the variation of luminance changes. Modifications to account for color changes, such as cDVS [34], implement events to indicate the increase or decrease in the measured average wavelength. Proposed by Moeys et al. [35], the SDAVIS is established on a DAVIS sensor with RGBW color filters to recover color change information. For each color, the DVS process is performed separately, so the sensor outputs color change events.

Moreover, a few works have proposed dichromatic or trichromatic color event sensors [36] based on stacked junctions. These sensors are not built around a Bayer filter or any other color mosaic because the photodiodes are stacked on top of each other. Thus, each pixel corresponds to a specific position and each component of the color is measured at that exact position.

Besides these new sensors, algorithms have been proposed to process event information for color reconstruction. Bajestani et al. [37] attempted to reconstruct color from monochromatic events and structured light. The SDAVIS [35] reconstructs pixel color from RGBW color filters. Marcireau et al. [13] have developed their own color sensor using ATIS sensors with beam splitters for color separation. One ATIS operates independently for each color channel. Therefore, the resulting events contain color information that updates whenever one of the three RGB sensors is triggered.

This color information can be utilized for color segmentation and object classification. The authors of [13] proposed an algorithm to track uniformly colored objects, which is similar to our case study. To estimate the color of an event and the object it belongs to, they use bivariate normal probability distributions. Extracting the color signature of each tracked object requires prior color calibration.

The present work has two objectives: first, to accurately reconstruct the color from the event stream by using event information, and second, to classify the colors of objects within a predefined palette.

### 7.2. Algorithm

This section presents a method for estimating the color of an object directly from the color events. A discretization of the colors in all incoming events based on the object’s color signatures is employed. This prior knowledge results in improved accuracy. Then, the current limitations of this method are discussed.

#### 7.2.1. Direct Estimation

Each event’s color corresponds to a 3D point in the YCbCr coordinate space. Since the events occur mainly on the contour of an object, a sensor’s pixel samples part of the background and part of the object. Hence, an event color ranges linearly from the scene background color to the object color. This means that the color estimation of the cluster is relative to the scene background. Considering a locally uniform and monochromatic background, the color of the background can be extracted from the noise events in the scene. That is, events that are not due to an object moving in the scene.

The estimated color is a mixture of the background and the object color.

(7) $$\begin{array}{cc} Y_{MS} & =pY_{obj}+\left(1-p\right)Y_{BG} \end{array}$$

(8) $$\begin{array}{cc} Y_{MS} & \propto Y_{obj} \end{array}$$

(9) $$\begin{array}{cc} Y_{MS} & \propto Y_{BG} \end{array}$$

At the arrival of each event, the following formulas are applied for each color component of the cluster. They are based on a mean-shift estimation of the color and apply a correction to compensate for part of the background.

(10) $$\begin{array}{cc} {\hat{Y}}_{MS,n} & =\alpha_{lum}{\hat{Y}}_{MS,n-1}+\left(1-\alpha_{lum}\right)e_{lum} \end{array}$$

(11) $$\begin{array}{cc} {\hat{Y}}_{obj,n} & =\frac{{\hat{Y}}_{MS,n}-Y_{BG}}{p}+Y_{BG} \end{array}$$

where ${\hat{Y}}_{MS,n}$ is the cluster’s luminance, estimated at the arrival of the event *n* using the mean-shift approach; $e_{lum}$ is the luminance of the event *n*; p∈[0,1] is a correction factor to compensate for the part of the BG; ${\hat{Y}}_{obj,n}$ is the estimated luminance value of the object; and $Y_{BG}$ is the luminance of the background.

The first Equation (10) calculates the influence of the new event on the estimated luminance, and the second Equation (11) corrects the influence of the background using the parameter *p* of (7). Ideally, *p* should be close to 1, but in most applications it is lower, depending on the contour of objects and uniformity of colors.

However, this method has limitations in certain contexts. Firstly, the calculated color is dependent on the background’s color, as the mixture is only an approximation. This technique is accurate when the background is uniform and monochromatic, analogous to the *Traffic* case study and the research conducted by Marcireau et al. [13]. Secondly, the computed color is susceptible to noise and the object specularity. Each event impacts the estimation, introducing imprecision into the outcomes due to variance. While this technique may not differentiate between shades for monochromatic objects, it is sufficient for distinguishing colors.

#### 7.2.2. Color Class

To alleviate this problem, and because color estimation is a challenging problem, we assume that each color can be assigned to a predefined color class. The palette of color classes used for the experiments is composed of colors extracted from the original video. This palette is considered prior knowledge.

By discretizing the colors, the goal is not to estimate the exact color of the objects, but rather to obtain the distribution of the color classes in the clusters, i.e., the relative proportion of each class. For each event assigned to a cluster, the corresponding color class of the event is selected and updated in the color distribution of the cluster, which counts the number of events of each color class.

This method is beneficial because it represents the various colors of the object in the distribution, and also considers the proportion of background events. This enables the tracking of complex objects with multiple colors in a complex background with the condition of the foreground and background colors being distinguishable. No prior scene-specific information is necessary besides defining the color palette. In fact, the color classes can be estimated using a different instance of clustering, known as a dynamic color palette. This results in a dynamic clustering of event colors that generates the principal color classes.

### 7.3. Experiments and Results

To estimate color, it is represented in the YCbCr format using 8-bit integers ranging from 0 to 255. The RMSE assesses the difference between the estimated color and the annotated object color. Separate RMSE values are calculated for each component, that is, the mean luminance error and the mean chrominance error. Again, a normalized error is given using the mean true value. The single object of *Pedestrian* is classified among a palette of blue, red, pink, green, black, gray colors in a total of eight classes. The classification accuracy is determined by dividing the number of correct evaluations by the total number of evaluations performed during the simulation. Again, the parameters are optimized on a video segment using hyperopt [33]. The optimized parameters of each scenario are presented in Table 6, and the estimation results are shown in Table 7.

The values of the parameters α indicate the rate of change for both luminance and chrominance. Higher values correspond to a lesser degree of variation in the value. The reason the parameter *p* is low for *Pedestrian* is the object’s changing contour, multiple colors, and textures.

**Figure 9:**
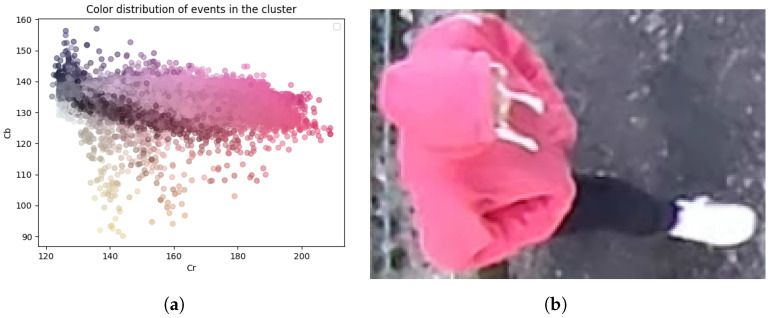
Color scattering for *Pedestrian*: (**a**) scatter plot, with each point corresponding to an event; (**b**) exemplary content of the cluster.

**Figure 10:**
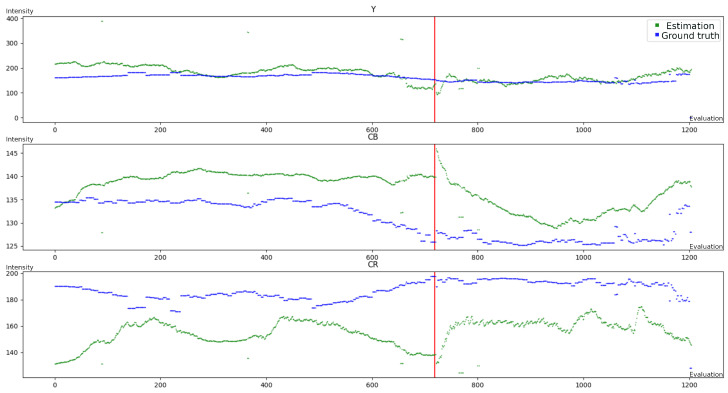
Color estimation for *Pedestrian*: (**top**) luminance, (**middle**) chrominance Cb, (**bottom**) chrominance Cr; the estimation is shown in green, and the ground truth in blue. The red line indicates the object’s exit and re-entrance.

**Figure 11:**
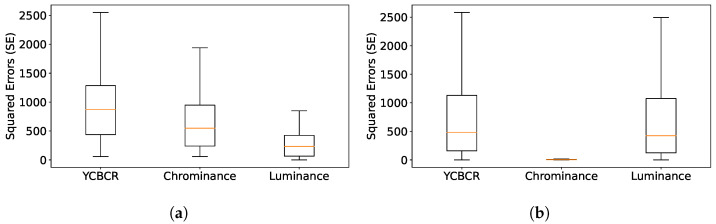
Box plots for color errors: (**a**) Pedestrian; (**b**) Traffic.

As mentioned above, estimating the object’s color directly from the event’s color gives bad results in the case of multicolored objects. Figure 9 illustrates this problem. It shows the color distribution of events during the experiment. On the right, the tracked object is represented, and on the left, there is a scatter plot where each point corresponds to the color of an event. One can recognize the color of the sweatshirt (pink), the color of the background (shades of gray), and the color of the skin (shades of beige). Furthermore, the range of pink extends between the background color and the expected object color. From this scatter, the algorithm estimates one spatio-temporal mean color that does not accurately represent the color distribution. This non-uniform object color induces a bias for the chrominance (see Figure 10). In the case of *Traffic*, the luminance varies a lot in the scene, but the chrominance can be estimated with a small error (2%). Our conclusion is that the reflective surfaces of the cars in the scene make it hard to precisely estimate the luminance. These two problems are reflected in the box plots in Figure 11; the chrominance errors are comparable to the luminance errors for *Pedestrian* (see Figure 11a), and chrominance errors do not contribute much to the color errors in *Traffic* (see Figure 11b). Notice that the errors are normalized differently for luminance and chrominance.

Generally, chrominance temporal variations tend to be minimal during experiments, as the object’s color remains relatively stable. However, luminance variations tend to be more substantial due to differences in the amount of light that shines on the objects. Therefore, the classification accuracy is generally high, particularly in the *Pedestrian* dataset, due to consistent lighting conditions and fewer reflective surfaces. However, when it comes to *Traffic*, accurately classifying shades of gray between white and black poses a challenge, even with correct classification of colored vehicles. The classification directly from the event stream yields an accuracy of 79.2%, while the use of the color palette results in a 92.3% accuracy. It is possible for gray to be misclassified as white or black based on shining light, given a 17% error in luminance.

In contrast to Marcireau et al. [13], we believe that spatio-temporal clustering achieves better tracking performance (see the position error in both scenarios), particularly in applications with non-uniform, textured, and multicolored backgrounds and objects. Thus, we advocate for the integration of color estimation in the cluster. However, in both works, the predefined palette should be replaced with dynamic color clustering for a wider range of applications that require less prior knowledge of the scene.

This section’s concludes with a few remarks. To achieve accurate estimation, the color information regarding the event needs to be corrected. Using a color signature or a cluster color provides more reliable results than using the color of a single event. Moreover, the color distribution in a cluster significantly improves classification performance. We conclude that the estimation method works well enough to classify objects of different colors, but gray shades cannot be accurately distinguished due to the imprecision of the luminance estimation (due to reflective surfaces in the scene). In addition, the use of color signatures can improve the accuracy of the method at the cost of more computations.

## 8. Conclusions

The polarity and intensity information retrievable with the state-of-the-art EBIS is neglected by most event-based image sensor algorithms. This work suggests using this enriched event stream to estimate object velocity and color for object tracking purposes. To accomplish this, a custom simulator was built by integrating a model of an advanced event-based image sensor. The constructed simulator allows testing of clustering and estimation algorithms using conventional videos and validating on annotated datasets. The first scenario follows a pedestrian walking on a textured background, and the second scenario shows two lanes of differently colored driving cars. Results are presented for these scenarios as they are comprehensible and annotated. Nevertheless, comparable outcomes were measured on other test cases.

Two practical methods for estimating velocity are proposed: the first computes velocity periodically, resulting in a more stable estimation but slower convergence; the second approach achieves faster convergence by updating velocity at each new event, but increases the sensitivity to noise. Both methods involve a trade-off between accuracy and responsiveness through their parameters. For the case studied, both methods provide satisfactory results. Moreover, this estimation can be utilized to enhance clustering by filtering noise based on velocity or predicting objects leaving the scene. However, the methods are demonstrated for apparent motion and would require an extra translation step to estimate real motion. The algorithm’s complexity is deliberately constrained, but its performance could be significantly improved by taking the object contour into consideration.

The direct color estimation from the event stream produces satisfactory results for the accurate estimation of object color. Nevertheless, when the luminance estimation is imprecise, this estimation confronts difficulties in classifying tones of the same color—for example, due to reflective surfaces. An improved description of cluster colors has been presented in the form of a discrete color distribution, which enhances the accuracy at the expense of more computations. In this instance, a predetermined color palette is employed for the color distribution, but another layer of clustering could be utilized to extract dynamic color signatures from the event stream.

Both velocity and color estimation are defined by tunable global parameters that could be replaced by dedicated parameters associated to each cluster and automatically tuned for each object and scenario.

The aim of this paper is to showcase the effectiveness of straightforward techniques in estimating features like velocity and color from the data stream of an event-based image sensor. Our findings suggest that the information from intensity measurement and multiple color channels is valuable in extracting complex features from the scene without high-level representation of the events’ information. Potential applications encompass optical flow estimation, motion segmentation, color segmentation, and object recognition, among others.

## Figures and Tables

**Table 1:** Video properties: the object exit indicates whether or not an object leaves the scene during the scenario.

Video Name	Number of Frames	Frame Rate	Frame Resolution	Number of Events	Number of Objects	Object Exit
Pedestrian	277	10 fps	270×480	80,135	1	Yes
Traffic	291	20 fps	480×270	277,951	12	Yes

**Table 2:** Color classes in *Traffic* (ground truth).

**Color Class**	Red	Blue	Yellow	White	Gray	Black
**Number**	1	1	1	5	2	2
**Color**	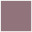	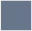	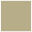	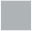	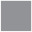	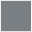

**Table 3:** Attributes of a cluster.

Attributes	Symbolic Representation
Position (center)	$P=(P_{x},P_{y})$
Size	$R=(R_{x},R_{y})$
Activity	*A*

**Table 4:** Parameters for velocity estimation.

Parameters		Pedestrian	Traffic
MCM	$\alpha_{vel,MS}$	$1-(4.34\times 10^{-4})$	$1-(8.3\times 10^{-5})$
	γ	152	698
Interval	$\alpha_{vel,Int}$	0.89	0.86
	τ [s]	0.11	0.15

**Table 5:** Measurements of velocity estimation errors.

Measures		Pedestrian	Traffic
Ground Truth Range		[−1.0,3.7]×[−7.6,5.7]	[−5.8,0.09]×[−7,8.2]
MCM	RMSE [pixel/$T_{frame}$]	1.498	2.6
	NRMSE	18.1%	24.5%
	Angular	17.2°	15.1°
Interval	RMSE [pixel/$T_{frame}$]	1.08	2.1
	NRMSE	13.1%	19.8%
	Angular	18.6°	17.4°

**Table 6:** Parameters for color estimation.

Parameters	Pedestrian	Traffic
$\alpha_{lum}$	$1-(9.2\times 10^{-5})$	0.999
$\alpha_{chro}$	0.9986	0.992
*p*	0.33	1

**Table 7:** Measurements of color estimation errors.

Measures		Pedestrian	Traffic
Position	RMSE [pix]	3.74	3.49
YCbCr	RMSE	35.2	25.9
	NRMSE	12.6%	11%
Luminance	RMSE	19.85	25.7
	NRMSE	12.3%	17.5%
Chrominance	RMSE	29.1	3.4
	NRMSE	12.7%	2%
Classification		93.75%/100%	79.2%/92.3%

## Data Availability

Data are contained within the article.

## Abbreviations

The following abbreviations are used in this manuscript:

EBIS	Event-based image sensor
DVS	Dynamic vision sensor
ATIS	Asynchronous time-based image sensor
DAVIS	Dynamic and active pixel vision sensor
APS	Active pixel sensor
TFS	Time to first spike
TDC	Time to digital converter
RGB	Red–green–blue
MCM	Mean center motion
RMSE	Root-mean-square error
MSE	Mean squared error
MS	Mean-shift
GT	Ground truth
